# Pickering emulsions stabilized by colloidal gel particles complexed or conjugated with biopolymers to enhance bioaccessibility and cellular uptake of curcumin

**DOI:** 10.1016/j.crfs.2020.05.001

**Published:** 2020-05-13

**Authors:** Andrea Araiza-Calahorra, Yunqing Wang, Christine Boesch, Yansheng Zhao, Anwesha Sarkar

**Affiliations:** a Food Colloids and Bioprocessing Group, School of Food Science and Nutrition, University of Leeds, Leeds, LS2 9JT, UK; b Nutritional Sciences and Epidemiology Group, School of Food Science and Nutrition, University of Leeds, Leeds, LS2 9JT, UK; c School of Food and Biological Engineering, Jiangsu University, Zhenjiang, 212013, China

**Keywords:** Pickering emulsion, Microgel, Curcumin, *in vitro* digestion, Caco-2 cells, Cellular uptake

## Abstract

The aim of this study was to investigate the fate of curcumin (CUR)-loaded Pickering emulsions with complex interfaces during *in vitro* gastrointestinal transit and test the efficacy of such emulsions on improving the bioaccessibility and cellular uptake of CUR. CUR-loaded Pickering emulsions tested were whey protein nanogel particle-stabilized Pickering emulsions (CUR-E_WPN_) and emulsions displaying complex interfaces included 1) layer-by-layer dextran sulphate-coated nanogel-stabilized Pickering emulsions (CUR-DxS+E_WPN_) and 2) protein+dextran-conjugated microgel-stabilized Pickering emulsions (CUR-E_WPDxM_). The hypothesis was that the presence of complex interfacial material at the droplet surface would provide better protection to the droplets against physiological degradation, particularly under gastric conditions and thus, improve the delivery of CUR to Caco-2 intestinal cells. The emulsions were characterized using droplet sizing, apparent viscosity, confocal and cryo-scanning electron microscopy, zeta-potential, lipid digestion kinetics, bioaccessibility of CUR as well as cell viability and uptake by Caco-2 cells. Emulsion droplets with modified to complex interfacial composition (*i.e.* CUR-DxS+E_WPN_ and CUR-E_WPDxM_) provided enhanced kinetic stability to the Pickering emulsion droplets against coalescence in the gastric regime as compared to droplets having unmodified interface (*i.e.* CUR-E_WPN_), whereas droplet coalescence occurred in intestinal conditions irrespective of the initial interfacial materials. A similar rate and extent of free fatty acid release occurred in all the emulsions during intestinal digestion (*p > 0.05*), which correlated with the bioaccessibility of CUR. Striking, CUR-DxS+E_WPN_ and CUR-E_WPDxM_ significantly improved cellular CUR uptake as compared to CUR-E_WPN_ (*p < 0.05*). These results highlight a promising new strategy of designing gastric-stable Pickering emulsions with complex interfaces to improve the delivery of lipophilic bioactive compounds to the cells for the future design of functional foods.

## Highlights

• Curcumin (CUR)-loaded Pickering emulsion (PE) were designed with complex interfaces.
• Interfaces were protein nanogels, polymer-coated nanogels and conjugate-microgels.
• Conjugate-microgel (CM) and polymer-coated nanogel (PN) generated gastric-stable PE.
• Free fatty acid release and bioaccessibility of CUR were similar across the PEs.
• PEs with complex interfaces (CM and PN) effectively delivered CUR to Caco-2 cells.

## Graphical abstract

**Figure:**
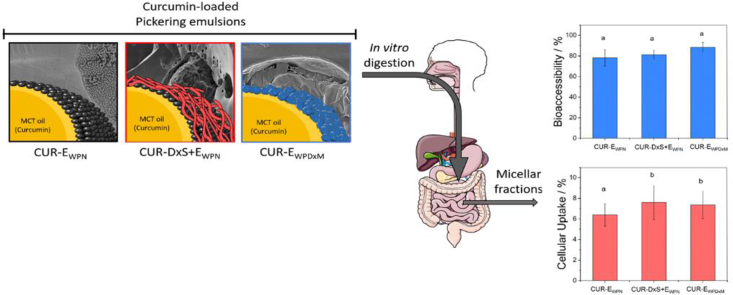

## 1 Introduction

Curcumin (CUR), a natural polyphenol, is the major curcuminoid (70–80%) present in the rhizomes of turmeric plant *Curcuma longa* (Goel et al., 2008). Due to its potential health-promoting properties such as antitumor, anti-oxidant, anti-microbial and anti-inflammatory, the incorporation of CUR into functional foods has been of major interest in recent years to both functional food and pharmaceutical industries (Anand et al., 2007). However, significant research challenges exist with the incorporation of CUR and its use as a bioactive ingredient due to limited aqueous solubility, high rate of metabolism and low bioavailability with rapid clearance of CUR (Tønnesen et al., 2002).

To address these delivery challenges of CUR, several colloidal approaches such as liposomes, vesicles, protein-based complexes, and emulsion-based delivery systems have surfaced in the literature (Amani et al., 2019, Araiza-Calahorra et al., 2018, Kolter et al., 2019, Sarkar and Mackie, 2020). In particular, emulsion-based delivery systems have been increasingly used to encapsulate CUR due to their simple processing technique, the fact that they can be made entirely from bio-based materials, and that they are suitable for incorporation into a variety of food matrices (Kharat and McClements, 2019). Nonetheless, emulsion-based delivery vehicles are mostly designed in isolation and often, the fate of the encapsulated CUR within these delivery systems during physiological transit remains poorly understood. In particular, the bioaccessibility of CUR after passing through the gastrointestinal (GI) tract and its permeability across the intestinal epithelium are crucial to understand the efficacy of these delivery vehicles, which have been given rare attention in the literature to date (Zou et al., 2015).

Among the emulsion-based delivery vehicles, Pickering emulsions have attracted significant recent scientific and industrial interests since they possess many advantages in terms of high stability against coalescence and Ostwald ripening (Rayner et al., 2012, Tzoumaki et al., 2011), and controlled digestibility of lipids by preventing competitive displacement by bio-surfactants (bile salts) (Dickinson, 2012, Ruiz-Rodriguez et al., 2014, Sarkar et al., 2016, Sarkar et al., 2019, Shimoni et al., 2013, Xiao et al., 2015). More specifically, Pickering emulsions stabilized by a wide range of particles, such as protein nanogels, modified starch granules, chitosan-tripolyphosphate complexes, silica, kafirin, ovotransferrin fibrils, nanocellulose and kaolinite (Araiza-Calahorra and Sarkar, 2019a, Asabuwa Ngwabebhoh et al., 2018, Lu et al., 2019, Marefati et al., 2017, Shah et al., 2016a, Shah et al., 2016b, Tang et al., 2019, Tikekar et al., 2013, Wei et al., 2019) have been recently used as delivery vehicles for CUR. However, only a few studies have investigated the biofunctionalities of the encapsulated CUR in these Pickering emulsions after *in vitro* digestion (Lu et al., 2019, Marefati et al., 2017, Shah et al., 2016b, Tikekar et al., 2013, Wei et al., 2019). Many of these emulsions have been prepared using inorganic particles, restricting their application in edible formats (Asabuwa Ngwabebhoh et al., 2018, Shah et al., 2016a, Shah et al., 2016b, Tang et al., 2019, Tikekar et al., 2013). In addition, literature is scarce on CUR bioaccessibility and potential cell toxicity as well as uptake of CUR by the cells when CUR is encapsulated in such Pickering emulsions (Lu et al., 2019).

In our previous study, we demonstrated the capacity of whey protein nanogel-stabilized Pickering emulsions to encapsulate CUR under different conditions of physiologically relevant different pHs and ionic strengths (Araiza-Calahorra and Sarkar, 2019a). In addition, Pickering emulsions stabilized by complex interfaces such as dextran sulphate (DxS)-coated nanogel particles (Araiza-Calahorra and Sarkar, 2019b) or conjugate microgels in which dextran (Dx) was covalently conjugated to protein before the micro-gelation process (Araiza-Calahorra et al., 2020) have successfully demonstrated higher kinetic stability to coalescence in the gastric phase as compared to that of non-modified simple nanogel-stabilized emulsions. The aim of this work was therefore to compare Pickering emulsions with complex interfaces (electrostatically-driven protein gel particles + biopolymer or covalently-conjugated protein-biopolymer gel particles at the interfaces) over nanogel particles as delivery vehicles for CUR for the first time. To test the efficacy of these delivery vehicles for curcumin, the fate of these Pickering emulsions loaded with CUR in simulated *in vitro* gastrointestinal digestion environment was investigated followed by assessment of curcumin bioaccessibility and cellular uptake. To our knowledge, the bioaccessibility and efficacy for delivering CUR to Caco2-cells after *in vitro* static simulated digestion when encapsulated in complex particle-stabilized Pickering emulsions has not been studied to date. Our hypothesis was that complex interfacial material can provide a better barrier to the droplets in the gastric environment and thus allow efficient release in the intestinal phase and therefore enhance the cellular uptake of CUR. Thus, novel insights from this study would advance the fundamental understanding of how interfacial design of emulsions can be tailored to alter gastrointestinal release and increase intestinal uptake of CUR.

## 2 Materials and methods

### 2.1 Materials

Curcumin (CUR) (1,7-bis(4-hydroxy-3-methoxyphenyl)-1,6-heptadiene-3,5-dione) (≥65% purity), dextran (Dx) as well as dextran sulphate (DxS) of molecular weight (MW) 500 kDa were purchased from Sigma-Aldrich Company Ltd (Dorset, UK) and used without any further purification. Powdered whey protein isolate (WPI) with ≥90% protein content was a kind gift from Fonterra Co-operative Group Limited (Auckland, New Zealand). Miglyol® 812 medium-chain triglyceride (MCT) oil with a density of 945 kg m^3^ at 20 °C was purchased from Cremer Oleo GmbH & Co (Germany) and was used as the dispersed phase without any further purification. All enzymes *i.e.* porcine pepsin (P7000, 526 U mg^−1^ using haemoglobin as a substrate), porcine pancreatin (P7545, 8 × USP and trypsin activity of 6.48 U mg^−1^ using TAME, N-p-Tosyl-L-arginine methyl ester hydrochloride, as a substrate) and porcine bile extract B8631 (total bile salt content 49 wt% with 10–15% glycodeoxycholic acid, 3–9% taurodeoxycholic acid, 0.5–7% deoxycholic acid, 5 wt% phospholipids) were purchased from Sigma-Aldrich Company Ltd. For cell culture experiments, human colon adenocarcinoma (Caco-2) cells were purchased from the European Collection of Authenticated Cell Culture (ECACC). Cell culture media and supplements *i.e.* Dulbecco's Modified Eagle Medium (DMEM), fetal bovine serum (FBS), Dulbecco's Phosphate-Buffered Saline (DPBS), non-essential amino acids (NEAA), trypsin EDTA, and penicillin-streptomycin mixture (5000 U mL-1)) were obtained from Gibco Cell Culture Products, Thermo Fisher Scientific (UK). Neutral red powder, HPLC-grade methanol, ethanol, and analytical-grade glacial acid, were acquired from Sigma-Aldrich Company Ltd. All solutions were prepared with Milli-Q water (resistivity of 18.2 MΩ cm at 25 °C) (Milli-Q apparatus, Millipore, Bedford, UK).

### 2.2 Methods

#### 2.2.1 Preparation of CUR-loaded Pickering emulsion systems

##### 2.2.1.1 Preparation of whey protein nanogel particles (WPN) and whey protein isolate+dextran conjugate microgel particles (WPDxM)

Whey protein nanogel particles (WPN) were produced based on a previously developed top-down technique (Araiza-Calahorra and Sarkar, 2019a). Briefly, WPI powder (10 wt%) was dissolved in 20 mM phosphate buffer at pH 7.0 for 2 h and the solution was heated in a temperature-controlled water bath at 90 °C for 30 min to form a heat-set gel (quiescent). The resultant WPI gels were pre-homogenized with phosphate buffer (5 wt% protein) using a hand blender (HB724, Kenwood) for 1 min and the resulting whey protein macrogel dispersion (5 wt% protein) was passed through a high-pressure homogenizer at 300 bars for two passes to create WPN. The resultant WPN was diluted with buffer to the desired protein concentration for the Pickering emulsion preparation.

Whey protein isolate+Dx conjugate powder was prepared as described previously (Araiza-Calahorra et al., 2020). The pH of the WPI+Dx solution (1:2 w/w ratio) was adjusted to pH 7.0 and gently stirred for 2 h at 25 °C. The WPI+Dx solution was stored at 4 °C overnight and then frozen at −20 °C for 6 h. Samples were then freeze-dried for 24 h and Maillard reaction of the resulting WPI+Dx was promoted by incubating the powder in a pre-heated desiccator at 60 °C for 24 h, with relative humidity (79%) controlled by saturated KBr solution.

Whey protein isolate+Dx conjugated microgels (WPDxM) with a degree of conjugation of 10% were produced using the aforementioned method used for creating WPN with minor modifications. Briefly, the conjugate powder was dispersed for 2 h in phosphate buffer at pH 7.0 to ensure complete dissolution to a final protein concentration of 11.6 wt%. The conjugate solution was heated in a temperature-controlled water bath at 65 °C for 1 h to form a heat-set gel (quiescent), followed by cooling down for 15 min and stored at 4 °C. The obtained gels were pre-homogenized with phosphate buffer (2 wt%) to create macrogel particles using a hand blender (HB724, Kenwood) for 1 min and then passed through a high-pressure homogenizer at 300 bars twice to create microgel particles. Obtained conjugate microgel particles (WPDxM) were diluted with buffer to the desired protein concentration for the Pickering emulsion preparation.

##### 2.2.1.2 CUR-loaded Pickering oil-in-water emulsion preparation

The oil phase was prepared by dissolving 2 wt% CUR into heated MCT-oil (60 °C), by magnetically stirring for 30 min, and centrifuging for 10 min at 4 °C to remove any undissolved CUR (Araiza-Calahorra and Sarkar, 2019a). Oil-in-water Pickering emulsions (80:20 w/w) containing CUR *i.e.* CUR-E_WPN_ or CUR-E_WPDxM_ were prepared using WPN or WPDxM as Pickering stabilizers, respectively. Coarse emulsions were prepared by homogenizing the MCT-oil containing CUR with fresh WPN or WPDxM aqueous suspension at pH 7.0 (1 wt% final protein concentration in all emulsions) using an Ultra Turrax T25 homogenizer (IKA-Werke GmbH & Co., Staufen Germany) at 13, 500 rpm for 1 min. Fine CUR-E_WPN_ or CUR-E_WPDxM_ droplets were prepared by passing the coarse emulsions twice through a high-pressure homogenizer at 300 bars. For the biopolymer-coated Pickering emulsions, CUR-E_WPN_ (40 wt% MCT-oil) and aqueous dispersions of DxS of 500 kDa Mw (0.4 wt%) were mixed in 1: 1 w/w at pH 3.0 to allow mutually attractive interaction between the cationic WPN and anionic DxS at the interface, as previously described by Araiza-Calahorra and Sarkar (2019b) and produce CUR-DxS-E_WPN_ (20 wt% MCT, 1 wt% WPN). For comparison purposes, all emulsions contained the same amount of oil and protein.

#### 2.2.2 Particle and droplet size measurements

Light scattering was used to measure the size distribution of the initial nanogel/microgel particles (dynamic light scattering, DLS) and fresh emulsion droplets (static light scattering, SLS) undergoing *in vitro* gastrointestinal digestion. Aqueous dispersions of WPN and WPDxM was measured using DLS at 25 °C using a Zetasizer Nano-ZS (Malvern Instruments, Malvern UK) after 100 × dilution in phosphate buffer (pH 7.0) at room temperature. Droplet size distributions before and after the *in vitro* digestion of the emulsion samples were determined using SLS at 25 °C using Malvern MasterSizer 3000 (Malvern Instruments Ltd, Malvern, Worcestershire, UK). The mean particle size of the emulsions was reported as volume mean diameter (*d*_43_) as it is more sensitive to droplet aggregation with systems showing bimodal size distribution. Results are based on three measurements on triplicate samples.

#### 2.2.3 *ζ*-potential measurements

The *ζ*-potential values of aqueous dispersions of the nanogel and microgel particles and the three Pickering emulsion samples were determined using Zetasizer, Nano ZS series, Malvern Instruments, Worcestershire, UK. Samples before and after *in vitro* digestion were diluted to 0.01% particle or 0.004 wt% oil in 100 × in phosphate buffer (pH 7.0) or SGF buffer (pH 3.0) or SIF buffer (pH 7.0) depending upon the condition and added to a folded capillary cell (Model DTS 1070, Malvern Instruments Ltd., Worcestershire, UK). The *ζ*-potential measurements were performed for duplicate samples with three readings for each of them.

#### 2.2.4 Apparent viscosity

The apparent viscosity of the freshly prepared Pickering emulsions was measured using a rheometer (Kinexus Ultra+, Malvern Instruments Ltd, Worcestershire, UK) equipped with a cone-and-plate geometry (diameter 40 mm, model: CP4/40 SS017SS). About 1.4 mL of the emulsion sample was placed onto the sample plate. Apparent viscosities were obtained for all the emulsion samples as a function of shear rates ranging from 1 to 1000 s^−1^ at 37 °C. Data from the flow curves were fitted to Ostwald de Waele fit as shown in Eq. (1):

(1) $$\eta_{a}\left(\dot{\gamma }\right)=K{\dot{\gamma }}^{n-1}$$

where *η*_a_ is the apparent viscosity, $\dot{\gamma }$ is the shear rate, *K* is the consistency index and *n* is the flow behaviour index. Linear regression analysis was applied to the data to calculate the flow behaviour index and the consistency coefficient.

#### 2.2.5 In vitro gastrointestinal digestion (static model)

A static digestion model was used in the *in vitro* digestion experiment employing a slightly adapted version from Minekus et al. (2014) omitting the oral step. Exactly 5 mL of the CUR-loaded Pickering emulsions at pH 3.0 (pre-incubated at 37 °C, 1 h) were mixed with 5 mL of simulated gastric fluid (SGF), consisting of 0.257 g L^−1^ of KCl, 0.061 g L^−1^ of KH_2_PO_4_, 1.05 g L^−1^ of NaHCO_3_, 1.38 g L^−1^ of NaCl, 0.0122 g L^−1^ of MgCl_2_(H2O)_6_, 0.024 g L^−1^ of (NH_4_)2CO_3_ and 2000 U/mL pepsin at pH 3.0. The mixture was incubated for 2 h at 37 °C under agitation using a shaking water bath (Grant Instruments Ltd, Cambridge, UK) at 100 rpm.

To allow sequential gastrointestinal digestion, after 2 h of incubation, the pH of the sample + SGF (10 mL) was adjusted to pH 6.8 with 1 M NaOH and mixed with 7.73 mL of simulated intestinal fluid (SIF) electrolyte stock solution consisting of 0.254 g L^−1^ of KCl, 0.054 g L^−1^ of KH_2_PO_4_, 3.570 g L^−1^ of NaHCO_3_, 1.123 g L^−1^ of NaCl and 0.335 g L^−1^ of MgCl_2_(H2O)_6_, 1.25 mL fresh bile (10 mM in the final digesta), 20 μL of 0.3 M CaCl_2_ and 1 mL of a pancreatin solution (100 U/mL based on trypsin activity in the final digesta) made up in SIF electrolyte stock solution. The *in vitro* intestinal digestion was carried out over 3 h at pH 6.8 and 37 °C.

During this 5 h *in vitro* digestion period, samples (sample+SGF and sample+SGF+SIF) were periodically collected for characterization. Samples were also prepared where CUR was dispersed in MCT-oil without any added protein gel particles and without employing any emulsification process (no vehicle) and also digested using similar SGF and SIF buffer. To stop the pepsin activity at specific time points, 0.2 M sodium bicarbonate was added to the samples to reach a final pH of 7.0. The pancreatin activity was stopped by adding 0.1 M of 4-(2-aminoethyl)benzenesulfonyl fluoride hydrochloride (Pefabloc©) to the sample (5 mM final concentration). Experiments were performed in triplicate and mean values were calculated.

#### 2.2.6 Free fatty acid release

After passing through simulated gastric and intestinal conditions, the free fatty acids (FFAs) released from the CUR-loaded emulsions were measured by using an automatic pH-stat titration unit (TIM 856 titration manager, Titralab, Radiometer analytical). Noteworthy that for doing the pH stat analysis of FFA release, this was a separate experiment where no aliquots were removed during the sequential gastrointestinal digestion. The pH-stat was used to monitor and control the pH at pH 6.8 for 3 h. The volume of added NaOH (0.25 M) was assumed to be equal to the amount of free fatty acids generated by the lipolysis of emulsified triacylglycerols. The amount of free fatty acids released was calculated from the titration curves as described by Sarkar et al. (2016). Using a nonlinear regression model, the kinetic parameters for the initial stages of FFA release were derived as described previously (Sarkar et al., 2016, Sarkar et al., 2019) using Eqs. (2), (3).

(2) $$\Phi_{t}=\Phi_{max}\left[1-\exp\left(\frac{-6kM_{w}Dnt^{2}}{\rho_{o}d_{0}^{2}{\text{Γ}}^{max}}\right)\right]$$

where *t* is the lipid digestion time in the intestine (min), *Φ*_max_ is the maximum total FFA level (%), *k* (mol s^−1^ m^−2^) is the conversion rate of the lipid per unit area of the emulsion droplet surface, occurring at the maximum lipase surface coverage, *M*_*w*_ is the molecular weight of MCT-oil, *d*_o_ is the initial average diameter of the emulsions (*d*_32_) and *ρ*_o_ is the density of the MCT-oil. *Γ*^max^ is the maximum coverage of the surface by the enzyme, *D* is the diffusion coefficient of the enzyme in the continuous aqueous phase and *n* donates the molar concentration of the lipase in SIF solution. In addition, the lipolysis half time (*t*_1/2_) (minutes) *i.e.* the time required to achieve half of the maximum extent of lipid digestion was obtained from Eq. (3) (Sarkar et al., 2016, Sarkar et al., 2019):

(3) $$t_{1/2}=\text{ln}\left(2\right)\left(\frac{d_{o}\rho_{o}}{6kM_{w}}\right)$$

#### 2.2.7 Bioaccessibility of CUR

The bioaccessibility of CUR in the Pickering emulsions was determined after 5 h of sequential *in vitro* gastrointestinal digestion. The digesta obtained at the end of the sequential gastrointestinal digestion process was centrifuged at 3000×*g* for 50 min at 5 °C. The middle layer was considered to be the “micellar fraction”, in which the CUR was solubilized. The concentration of CUR in the micelles was analysed using high-performance liquid chromatography (HPLC) analysis. An Agilent 1200 series HPLC instrument coupled with DAD detector was used for the analyses of CUR. The measurement wavelength was 425 nm, and the separation column was Agilent XDS-C18 (150 mm×4.6 mm, 5μm). The mobile phase A was 0.2% of acetic acid aqueous, and the mobile phase B was acetonitrile, with a flow rate of 1 mL/min. The gradient elution program was: 0min, 60% of A; 4–10min: 20% of A. The column temperature was maintained at 25 °C. The injection volume was 20 μL, and an external CUR standard was used for quantitative analysis. A calibration curve was prepared with standard CUR in acetonitrile in concentrations ranging from 0.1 μM to 20.0 μM. Bioaccessibility (%) of CUR was calculated by dividing the amount of solubilized CUR in the micellar phase by the amount of CUR in the emulsion.

#### 2.2.8 Microstructural characterization

##### 2.2.8.1 Confocal scanning laser microscopy (CLSM)

The microstructure of the samples before and after *in vitro* digestion was imaged using a Zeiss LSM 700 CLSM (Carl Zeiss MicroImaging GmbH, Jena, Germany) confocal microscope using an oil immersion 63 × lens and the pinhole diameter maintained at 1 Airy Unit to filter out the majority of the light scattering. A stock solution of Fast Green (1 mg mL^─1^ in Milli-Q water) was used to stain the protein particles to a final concentration of 0.1 mg mL^─1^, which was excited at a wavelength of 633 nm. The emission filter was set at 660–710 nm. Samples were placed on a concave confocal microscope slide, secured with a glass coverslip and imaged.

##### 2.2.8.2 Cryogenic-scanning electron microscopy

Cryogenic scanning electron microscopy (cryo-SEM) of the fresh CUR-loaded emulsion samples *i.e.* CUR-E_WPN_, CUR-DxS+E_WPN_, and CUR-E_WPDxM_ were conducted. Cryo-SEM images were acquired using heptane as the dispersed rather than MCT oil to avoid interference by crystallization of oil during the freezing step as used in previous studies by Destribats et al. (2014) and Araiza-Calahorra and Sarkar (2019a). The CUR-E_WPN_, CUR-DxS+E_WPN_, and CUR-E_WPDxM_ were mounted on rivets attached to the sample stub. The samples were plunge-frozen in liquid nitrogen “slush” at −180 °C and then transferred to the cryo-preparation chamber in the SEM. The frozen Pickering emulsion droplets were cleaved and then etched at −95 °C for 4 min. Next, the samples were coated with 5 nm of platinum (Pt). Finally, the Pt-coated samples were transferred to the SEM for imaging at −135 °C. The heptane-based emulsion samples were imaged in a FEI Quanta 200 F ESEM with a Quorum Polar Prep 2000 cryo system.

#### 2.2.9 Cell-based assays

##### 2.2.9.1 Cell culture

Human colon adenocarcinoma cells, Caco-2, were cultivated in high glucose DMEM medium with pyruvate, supplemented with 10% FBS, 1% NEAA, 100 U mL^-1^ penicillin and 100 μg mL^-1^ streptomycin. Cells were grown under standard conditions at 37 °C with 5% CO_2_ in a humidified atmosphere and medium was changed every 2–3 days. Cells were used for experiments within 10 in-house passages.

##### 2.2.9.2 Cytotoxicity assay

The cytotoxicity of the micellar phases of the Pickering emulsions was assessed by the neutral red assay as described previously (Perez-Hernandez et al., 2020). Briefly, Caco-2 cells were seeded in 24-well plates at a density of 1 × 10^5^ cells cm^−2^ and, upon reaching min 90% confluence, they were treated with CUR dissolved in DMSO (0.5–6.0 μM), micellar phase of digested CUR-encapsulated emulsion sample or micellar phase of digested emulsion sample without CUR. The medium was removed after 2.5 h and replaced by DMEM containing 40 μg mL^−1^ neutral red dye which was incubated for 2 h. Subsequently, cells were washed with DPBS and the intracellular dye extracted by distain solution (AcOH/H_2_O/glacial acid, 50:49:1, v/v/v) for 10–15 min. Absorbance of the neutral red dye was measured at 540 nm using a microplate reader (Tecan Spark 10 M, Switzerland). Viability of the Caco-2 cells was calculated as the percentage of control cells (DMEM medium only). DMSO (5%) was included as control which lowered the cell viability to 67%. Experiments were conducted over three independent passages and performed in triplicates per experiment.

##### 2.2.9.3 Cellular curcumin uptake

The cellular uptake of CUR from the micellar phase of digested CUR-encapsulated Pickering emulsions was determined using HPLC. Caco-2 cells were seeded at a density of 2 × 10^6^ cells per 10 cm Petri dish. When reaching confluence (min 90%), the cells were exposed to different micellar phase digesta containing 1 μM CUR for 2 h under standard conditions. Subsequently, cells were washed twice with ice-cold DPBS and lysed with methanol. The cell pellets were collected and subjected to extraction, which involved vortex (1 min), sonication (4 °C, 5 min) and centrifugation (10,000×*g*, 4 °C, 10 min). The supernatant was filtered through a 0.2 μm PTFE syringe and prepared for the subsequent curcumin HPLC analysis. Experiments were conducted in triplicate in subsequent cell passages.

### 2.3 Statistical analysis

Significant differences between samples were determined by one-way ANOVA and multiple comparison test and Tukey’s adjustment was performed using SPSS software (IBM, SPSS statistics, version 24) and the level of confidence was 95%. Experiments were conducted at least in triplicate. Results in tables are expressed as mean ± standard deviation. Error bars in figures represent standard deviation.

## 3 Results and discussion

### 3.1 Characteristics of curcumin Pickering emulsions

**Fig. 1:**
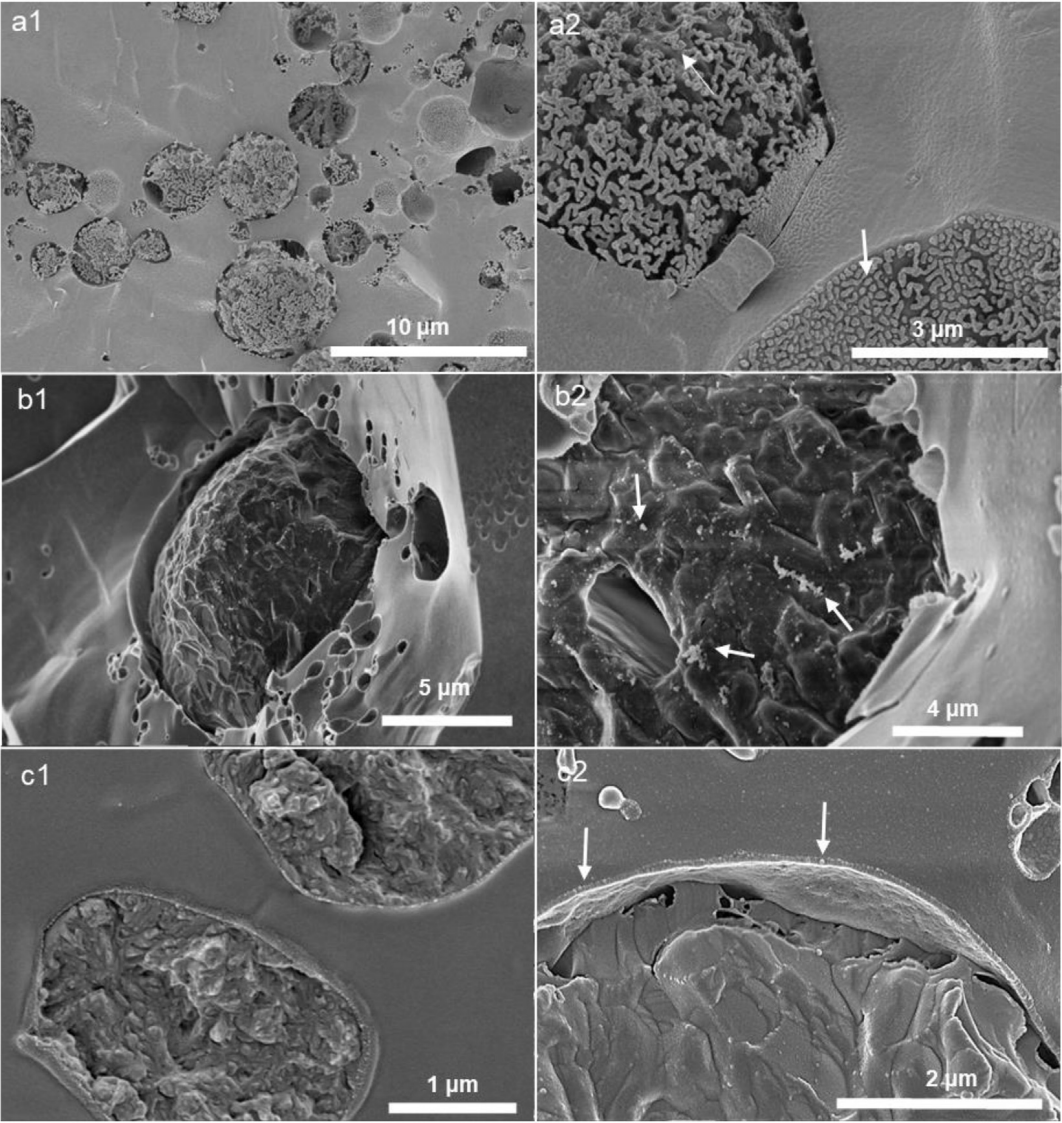
Cryo-SEM images of the three Pickering emulsions used for delivering curcumin i.e. a) CUR-EWPN emulsion (magnification of 15,000 × (a1), and 50,000 × (a2), respectively), b) CUR-DxS+EWPN (magnification of 25,000 × (b1), and 50,000 × (b2), respectively) and c) CUR-EWPDxM (magnification of 10,000 × (c1), and 20,000 × (c2), respectively). Arrows indicate the nanogel particle in a2, nanogel particle aggregated with dextran sulphate in b2, and conjugate microgel particles in c2.

Initially, we evaluated the characteristics of the Pickering emulsion systems (CUR-E_WPN_, CUR-DxS+E_WPN_, and CUR-E_WPDxM_) using droplet size, size distribution, microstructure, surface morphology, and electrical characteristics (*ζ*) before and after gastric and intestinal digestion steps. Apparent viscosity of the emulsions was also evaluated to understand how bulk properties might impact digestion behaviour. Fig. 1 shows the surface morphology of the freshly prepared Pickering emulsion samples with particle-laden interfaces probed using cryo-SEM. Fig. 1a1 shows several CUR-E_WPN_ emulsion droplets homogeneously distributed throughout the micrograph with a woolly jacket of WPN attached to the droplet surface giving a raspberry-like surface appearance. At higher magnification (Fig. 1a2), WPN seemed to have an end-to-end aggregation at the droplet surface, which might be associated with nanogel merging with each other at the interface. Besides, sample preparation process (e.g. freeze-fracturing) during the cryo-SEM might had also result in such aggregation. Similar morphology has been also previously observed in cryo-SEM images of Pickering droplets in WPN-stabilized oil droplets without containing CUR (Araiza-Calahorra and Sarkar 2019a). This suggested that the addition of CUR had limited effects on the surface morphology of the droplets.

Pickering emulsion droplets stabilized by a more complex interface *i.e.* with WPN electrostatically-coated with DxS of 500 kDa M_w_ were spherical with an average diameter of ~15 μm (Fig. 1b1). Looking at the surface of such droplets at higher magnification, it can be observed that individual spherical WPN seemed to be aggregated, which can be an effect of DxS coating electrostatically attracting multiple neighbouring WPN within a thread-like network (Fig. 1b2). The other Pickering emulsion with complex interface *i.e.* CUR-E_WPDxM_ samples showed the presence of conjugated microgel particles formed a thin surface layer adopting a more discrete configuration of individual microgel particles (Fig. 1c1 and c2).

**Fig. 2:**
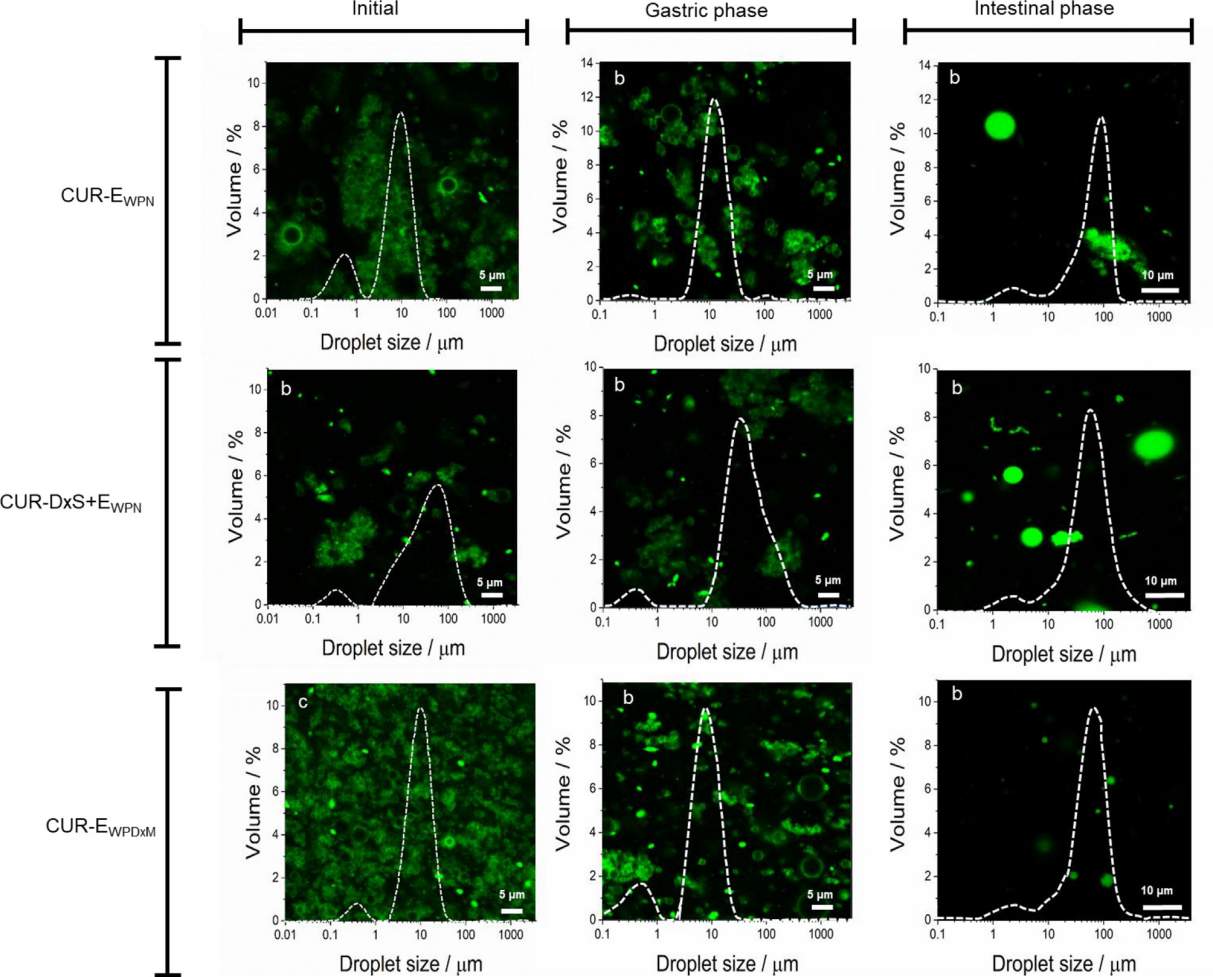
Confocal images with superimposed droplet size distribution of the freshly-prepared Pickering emulsions *i.e.* CUR-E_WPN_, CUR-DxS+E_WPN_ and CUR-E_WPDxM_ and after their exposure to 120 min of *in vitro* gastric or 180 min of *in vitro* sequential gastrointestinal digestion conditions.

The droplet size distribution and mean diameters with representative confocal images of the three freshly prepared Pickering emulsion systems are shown in Table 1 and Fig. 2. The initial droplet size distribution of the three emulsions, CUR-E_WPN,_ CUR-DxS+E_WPN_, and CUR-E_WPDxM_ presented bimodal distributions where the peak in the area of 0.1–1 μm in all systems correspond to unadsorbed particles in line with the systems previously studied without loaded CUR (Araiza-Calahorra and Sarkar, 2019b, Araiza-Calahorra and Sarkar, 2019a) and small emulsion droplets, while the peak in the area of 1–100 μm corresponds to the bigger emulsion droplets (Fig. 2). The CUR-E_WPN_ presented oil droplet ranging in size from 1 to 50 μm and an average droplet diameter (*d*_*43*_) of 14.93 μm (Table 1), whereas CUR-DxS+E_WPN_ system presented oil droplets ranging in size from 3 to 100 μm and an average droplet diameter (*d*_43_) of 54.56 μm (Table 1). This can be expected as the electrostatic coating with DxS resulted in droplet flocculation with DxS not only binding to individual droplets but also connecting two or more adjacent droplets as can be clearly observed as flocs in the confocal images (Fig. 2). On the other hand, the conjugated microgel-laden system *i.e.* CUR-E_WPDxM_ presented a *d*_43_ of 7.9 μm (Table 1). From the confocal images in Fig. 2, it is noticeable that all three systems had proteinaceous particles (stained in green) adsorbed at the interface acting as a barrier against oil droplet coalescence.

**Table 1:** Mean droplet size and ζ-potential of initial emulsions.

Emulsions	*d*_43_/μm	*ζ*-potential/mV
CUR-E_WPN_	14.93 ± 3.3	−26.9 ± 0.5
CUR-DxS+E_WPN_	54.56 ± 6.1	−32.33 ± 4.8^a^
CUR-E_WPDxM_	7.9 ± 0.2	−15.36 ± 1.1

a Note that the initial emulsion CUR-DxS+E_WPN_ was at pH 3.0 to allow electrostatic attraction between WPN and DxS, unlike the other two emulsions, which were at pH 7.0.

All the Pickering emulsions studied were negatively charged (Table 1). CUR-E_WPN_ presented a high negative charge because the initial pH of the emulsions was appreciably above the isoelectric point (*pI*) of the whey protein isolate (*pI* ~5.2), whereas the decreased magnitude of negative charge for CUR-E_WPDxM_, as compared to CUR-E_WPN_, may be attributed to the covalent attachment of the neutral dextran molecule (Araiza-Calahorra et al., 2020). For CUR-DxS+E_WPN_, the initial pH of the solution was at pH 3.0 to allowed electrostatic deposition of the dextran sulphate to the WPN-stabilized interface (Table 1). Hence, the negative charge of the droplets suggested that the negatively-charged biopolymer was successfully adsorbed onto the cationic WPN-stabilized oil droplets at pH 3.0 (Araiza-Calahorra and Sarkar, 2019b).

**Fig. 3:**
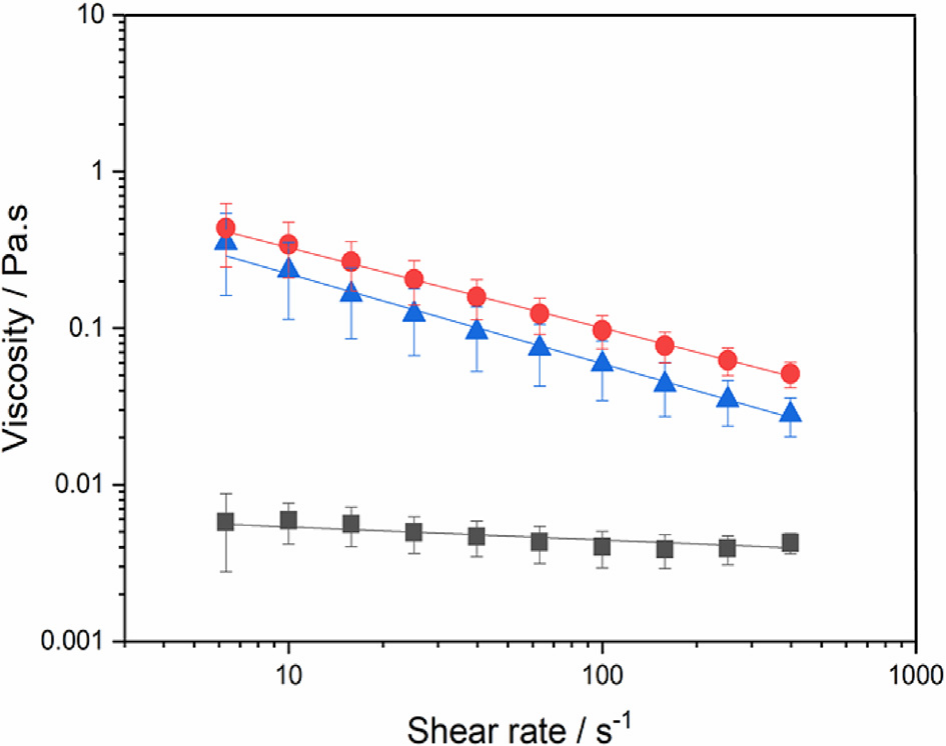
Flow curves of freshly prepared Pickering emulsions *i.e.* CUR-E_WPN_ (black squares) CUR-DxS+E_WPN_ (blue triangles) and CUR-E_WPDxM_ (red circles) at 37 °C. Data points represent the average of at least three measurements on triplicate sample. Error bars indicate the standard deviations. Solid lines are the best fits to the experimental data predicted using the Ostwald de Waele model (Eq. (1)). (For interpretation of the references to colour in this figure legend, the reader is referred to the Web version of this article.)

Fig. 3 shows the apparent viscosity (*η*_a_) versus the shear rate of the initial emulsions before *in vitro* digestion. The apparent viscosity decreased as the shear rate increased for the emulsion samples with complex interfaces and the shear-sweep data were satisfactorily fitted to the Ostwald de Waele model (R^2^ ranging from 0.993 to 0.997). In other words, CUR-DxS+E_WPN_ and CUR-E_WPDxM_ showed shear thinning behaviour, with flow behaviour index (*n*) ranging from 0.43 to 0.49, whilst CUR-E_WPN_ emulsions showed a Newtonian behaviour.

Additionally, the magnitude of *η*_a_ was orders of magnitude higher at a shear rate ranging from 1 to 100 s^−1^ for CUR-DxS+E_WPN_ and CUR-E_WPDXM_ samples as compared to CUR-E_WPN_, which suggested that the *η*_a_ increased with the addition of dextran by either conjugation or electrostatic complexation approaches. For CUR-DxS+E_WPN_, the presence of unbound high M_w_ DxS (500 kDa) remaining in the continuous phase might have increased the *η*_a_ of the emulsions. Also, the inter-droplet flocculation, as observed in the confocal micrograph of CUR-DxS+E_WPN_ (Fig. 2), might have contributed to the higher viscosity and eventual thinning when the flocs were broken down into individual droplets as a function of increasing shear rate to allow subsequent flow. Even for CUR-E_WPDxM_, the inter-droplet flocculation shown in Fig. 2 appeared to be the most plausible reason for such high shear-thinning behaviour where droplets aggregated owing to limited repulsive interactions (see zeta-potential values in Table 1). In summary, the *η*_a_ obtained for the systems containing dextran were not significantly different, while the *η*_a_ of CUR-E_WPN_ was significantly lower irrespective of the shear rates, which might play an important role in the degree of FFA release during the gastrointestinal conditions and consequently bioaccessibility and cellular uptake of CUR.

### 3.1 Characteristics of CUR-loaded Pickering emulsions during *in vitro* gastrointestinal digestion

**Fig. 4:**
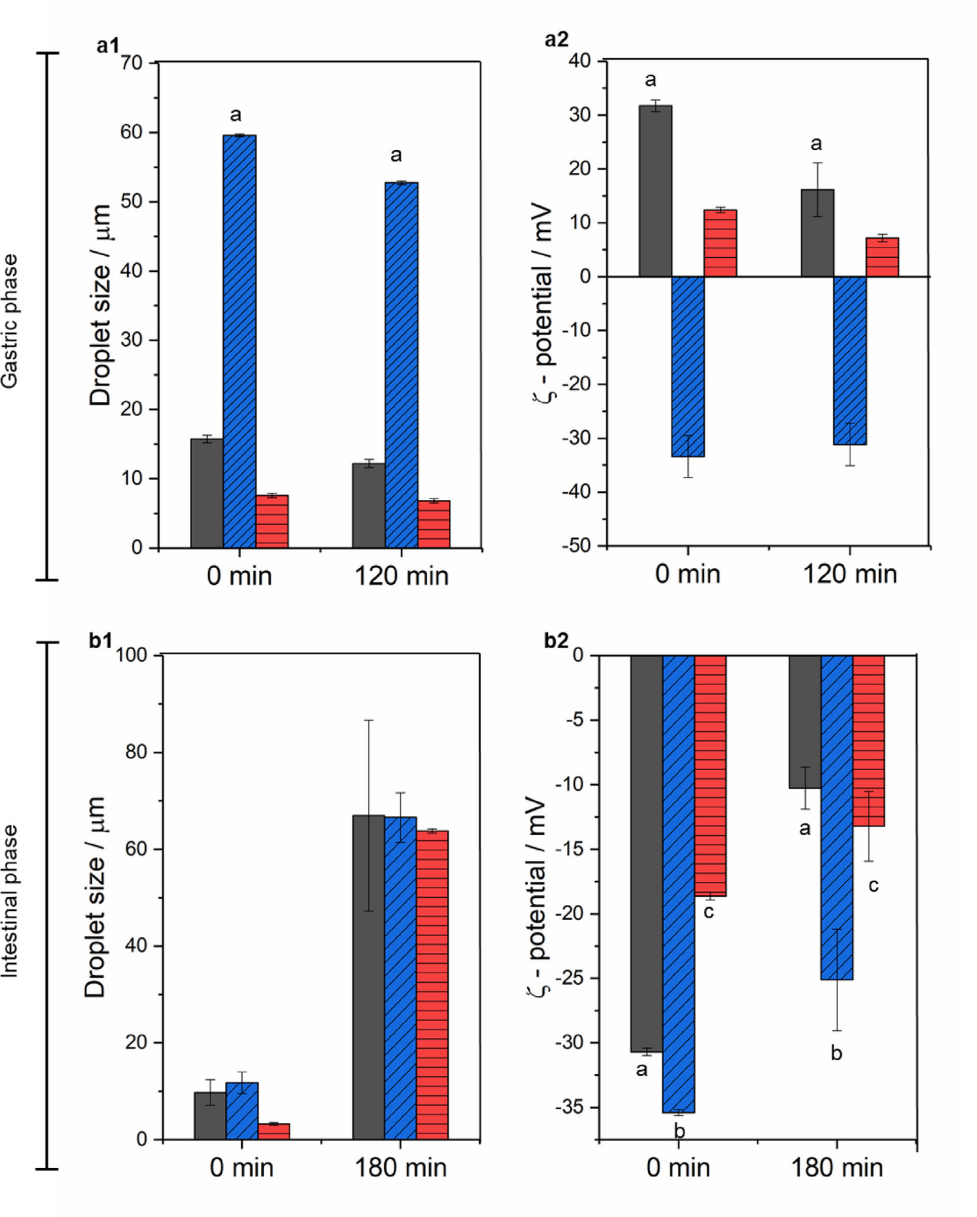
Average droplet diameter (*d*_43_) (1) and ζ-potential value (2) of CUR-EWPN (black bars), CUR-DxS+E_WPN_ (blue bars with diagonal lines) and CUR-E_WPDxM_ (red bars with horizontal lines) after in vitro gastric digestion (a) and *in vitro* intestinal digestion (b). 0 min in each case indicates the behaviour of the emulsions in presence of SGF (a) and SIF (b) buffer without any added enzymes. Error bars represent the standard deviations. (For interpretation of the references to colour in this figure legend, the reader is referred to the Web version of this article.)

The kinetic stability and responsiveness of the three Pickering emulsions was accessed at gastric conditions without pepsin (*i.e.* SGF without pepsin, 37 °C) (see time 0 min in Fig. 4). Droplet size distribution did not change and consequently, *d*_43_ of the systems remained similar to those of the freshly prepared samples (*d*_43_ 15.75, 59.6 and 7.60 μm for CUR-E_WPN_, CUR-DxS-E_WPN_, and CUR-E_WPM_, respectively) (Table 1), confirming there were no SGF-induced effects when no pepsin was employed. This phenomenon of stable emulsions after the addition of gastric buffer (SGF) without pepsin was supported by the *ζ*-potential measurements where both CUR-E_WPN_ and CUR-E_WPDxM_ presented a charge reversal from negative to positive due to the protonation of the ionizable groups as they move from above to below the isoelectric point (Fig. 4b). Interestingly, there were no significant differences in the *ζ*-potential of CUR-DxS+E_WPN_ after addition of gastric buffer (*p > 0.05*).

After being exposed to 120 min of *in vitro* gastric digestion stage in the presence of pepsin, *d*_43_ values significantly decreased (*p > 0.05*) for CUR-DxS+E_WPN_ from 59.6 μm to 52.75 μm, (Fig. 4a), which explained the slight shift to smaller values observed in the droplet distribution with the breakdown of the droplet flocs (Fig. 2). For CUR-E_WPN_, the droplet size distribution evidenced some coalescence phenomena, which is clearly shown by a rise in a third peak in the range of 100–1000 μm size range (Fig. 2), whereas the *d*_43_ and droplet size distribution remained unchanged for CUR-E_WPDxM_. This behaviour in CUR-E_WPN_ might be related to the peptic hydrolysis of the proteinaceous nanogel particles at the surface of the emulsion droplets, which might have caused some coalescence of the droplets, whereas CUR-E_WPDxM_ presented good physical stability after the gastric stage. The complex interfaces were indeed successful in providing gastric stability to the droplets. Such desirable results in the case of CUR-DxS+E_WPN_ and CUR-E_WPDxM_ might be attributed to the polysaccharide coating/conjugation, which restricted the access of pepsin to potential cleavage sites of the protein (Araiza-Calahorra et al., 2020, Araiza-Calahorra and Sarkar, 2019b). On the other hand, the high bulk viscosity of the CUR-DxS+E_WPN_ and CUR-E_WPDxM_ (Fig. 3) may have also hindered the diffusion of pepsin to the proteinaceous sides of the particle (Sarkar et al., 2017).

Upon subjecting the emulsions to gastric conditions with pepsin, the *ζ*-potential became less positive for all the samples, with CUR-E_WPN_ presenting significant differences from +31.7 to +16.1 mV after 120 min of gastric digestion (Fig. 4b). This reduction in the absolute magnitude of *ζ*-potential after *in vitro* gastric conditions further supported the pepsin-induced hydrolysis of WPN particles when absorbed at the E_WPN_ interface. However, such changes were not seen in the emulsions stabilized by complex interfaces supporting the confocal images and size distribution data (Fig. 2), highlighting the kinetic stability of these emulsions with complex interfaces to droplet coalescence in the gastric conditions.

Under small intestinal conditions, all samples studied exhibited a significant increase in the *d*_*43*_ ranging between 60.25 and 69.25 μm corresponding to droplet coalescence irrespective of the initial interfacial material (Fig. 2, Fig. 4c). From the microscopic analysis, it became clear that the larger droplets measured by laser diffraction corresponded to the coalesced oil droplets of similar size after 180 min of intestinal conditions (Fig. 2). Also, noteworthy that considerable amount of such coalesced oil droplets might have not been captured during confocal imaging due to migration of the oil to the top of the microscopic slide caused by the density gradient, which might explain why the images in the intestinal phase showed such lower number of droplets and largely empty regions, irrespective of the interfacial material.

Such increase of droplet diameter (Fig. 4c) was more likely caused by the digestion of lipids and proteins by pancreatic lipase and trypsin, respectively. In the intestinal phase, the particles at the droplet surface were most likely hydrolyzed by trypsin creating peptide residues or particle fragments at the interface, thus making them incapable of providing coalescence stability, which can be expected in case of ‘enzyme-responsive’ particle-laden interfaces (Sarkar et al., 2019). Particularly, one might expect CUR-DxS+E_WPN_ to behave similarly to E_WPN_ because at intestinal pH (pH 6.8), both DxS and WPN are negatively-charged hindering any electrostatic attraction of the DxS to the WPN-coated surface, and hence this particular complex interface *i.e.* the WPN+DxS did not even existed at near alkaline pH. To our surprise, even CUR-E_WPDxM_ behaved similarly to E_WPN_ highlighting that trypsin was somehow more capable of hydrolysing the proteinaceous parts of the conjugated microgel particles as compared to pepsin (Fig. 2). Also, the dilution occurring in the emulsions by the addition of SGF and SIF might have reduced any anticipated viscosity-induced benefits in these emulsions with complex interfaces (Fig. 3). Finally, the lipid digestion products such as free fatty acids (FFAs), monoglycerides and diglycerides were also not capable of forming viscoelastic films to provide stability to the droplets against coalescence (Salvia-Trujillo et al., 2013b, Sarkar et al., 2019, Singh and Sarkar, 2011, Torres et al., 2019). The electrical charges on the emulsion samples after the intestinal phase significantly decreased for all samples indirectly highlighting the presence of lipid digestion products such as mono- and/or di-glycerides and FFA at the droplet surface (Fig. 4d). In summary, CUR-DxS+E_WPN_ and CUR-E_WPDxM_ systems were more stable under gastric conditions, however, with the addition of the intestinal components (*i.e.* pancreatin, bile salts, CaCl_2_), all the Pickering systems behaved similarly irrespective of the initial interfacial material.

### 3.2 Influence of emulsion type on lipid digestion kinetics

**Fig. 5:**
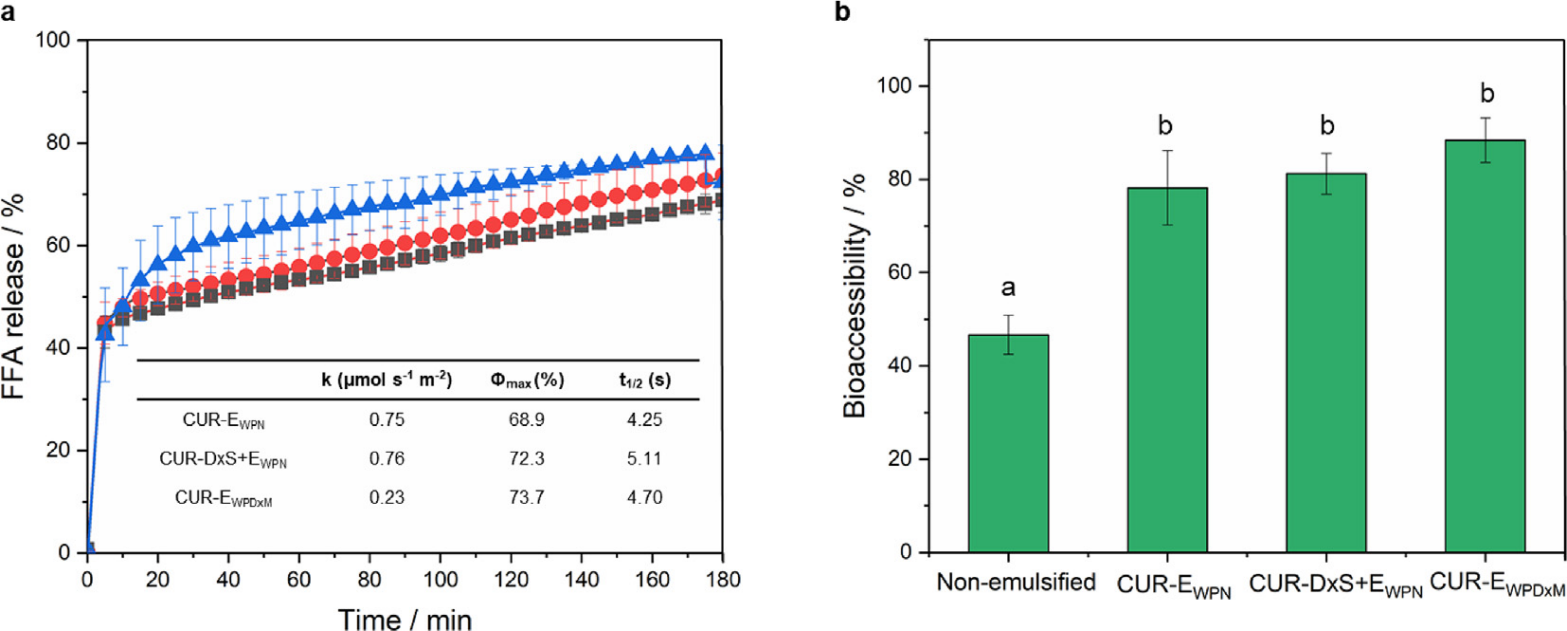
Percentage of free fatty acid (% FFA) released (a) from CUR-E_WPN_ (black squares), CUR-DxS+E_WPN_ (blue triangles) and CUR-E_WPDxM_ (red circles) with insets representing maximum FFA release (Φmax, %), lipolysis rate constant (*k*, μmol s^−1^ m^−2^) and the time to achieve 50% digestion (*t*_1/2_, s), and bioaccessibility (b) of CUR after *in vitro* gastrointestinal digestion from the micellar phase of the aforementioned emulsions. The solid lines connecting the data points in the %FFA cuves (a) are the best fits to the experimental data predicted using mathematical model (Eq. (2). Data presented are mean with standard deviation of three independent experiments. Different letters indicate significant differences. (For interpretation of the references to colour in this figure legend, the reader is referred to the Web version of this article.)

It was important to understand whether such gastric stability has any influence on the rate and degree of %FFA release as the latter is known to be related to the droplet size. All emulsions presented a steep increase in the amount of %FFA released within the first 5 min after exposure to neutral pH (pH 6.8), ions, bile salts, and pancreatin. It was followed by a more gradual increase at longer times until a relatively constant final value of %FFA was reached (Fig. 5a). Using Eqns (2), (3)), the time required for completion of 50% digestion (t_1/2_), and the digestion rate (*k*) were calculated (inset table in Fig. 5a). Interestingly, there was no significant difference between emulsion samples in terms of the rate (*k*) and extent (*Φ*_max_) of %FFA release (*p > 0.05*). This supports the droplet size results, where no significant difference in the final *d*_43_ between samples was observed in the intestinal stage, suggesting that the addition of dextran either by complexation or conjugation approaches did not influence the lipolysis rate and extent of the Pickering emulsions. However, the time required for 50% of digestion (−) was slight but significantly different between samples (*p < 0.05*). Similar results were reported on employment of dietary fibres on the digestion rate of emulsified lipids. A slight decrease in lipolysis rate with increasing concentrations of polysaccharides (chitosan, pectin or methylcellulose) was attributed to the interaction of the polysaccharides with digestion metabolites, such as bile salts, lipases, etc. (Espinal-Ruiz et al., 2014). Lipolysis of Pickering emulsions stabilized by lactoferrin nanoparticles electrostatically coated with iota-carrageenan rendered elevated rate and extent of lipolysis, whereas the use of alginate as a secondary coating significantly reduced both the afore-mentioned parameters (Meshulam and Lesmes, 2014). Changes in the emulsion lipolysis dynamics was explained by the physical changes in emulsion properties such as droplet size, and organization state (e.g. aggregation versus coalescence) due to the addition of the dietary fibre.

These results suggest that both, electrostatically or covalently attached dextran were able to control the initial rate of lipid digestion within a simulated *in vitro* system validating the hypothesis. However, the encapsulated lipid irrespective of the interfacial material were digested and released to the same extent.

### 3.3 Influence of emulsion type on CUR bioaccessibility

CUR bioaccessibility of the three CUR-loaded Pickering emulsions, as well as the non-emulsified MCT-oil are shown in Fig. 5b. It was found that curcumin bioaccessibility was 46.67 ± 4.19, 78.18 ± 7.97, 81.22 ± 4.38 and 88.42 ± 4.77% for CUR in non-emulsified MCT-oil, CUR-E_WPN_, CUR-DxS+E_WPN_, and CUR-E_WPDxM_, respectively. This suggests that CUR bioaccessibility increased when CUR was delivered in Pickering emulsion format in comparison to the non-emulsified bulk system. Between Pickering samples, the conjugation with Dx or the electrostatic deposition of DxS had no significant benefit over the WPN-stabilized emulsions (*p > 0.05*) on the bioaccessibility of CUR, which is in agreement with the FFA release results showing no significant difference (*p > 0.05*) (Fig. 5a). In addition, the *in vitro* bioaccessibility correlated positively with the total amount of FFAs produced at the end of the lipid digestion process of the three emulsion systems (Pearson correlation coefficient 0.981, *p* = 0.0182).

The lack of differences in the bioaccessibility (Fig. 5b) among the different delivery systems can be attributed to the fact that all of the emulsion systems had similar droplet characteristics (Fig. 2, Fig. 4) and consequently similar %FFA release (Fig. 5a) after the small intestinal digestion phase. In contrast, CUR in bulk MCT-oil (*i.e.* non-emulsified sample) presented significantly lower bioaccessibility under simulated intestinal conditions (*p < 0.05*) as compared to those of the emulsion counterparts (Fig. 5b). This result suggests that there was a more efficient transfer of CUR into the mixed micelles when incorporated into a Pickering emulsion-based delivery vehicle. The reduced surface area of the bulk oil prevented efficient access of the triglyceride to the lipase. Thus, the CUR remained dissolved within this oil phase and was not extracted effectively into the micellar phase reducing the bioaccessibility (Salvia-Trujillo et al., 2013a).

Previous studies on Pickering emulsions for CUR delivery have reported a bioaccessibility of 8.8% for kafirin-, 21% and 53% for chitosan tripolyphosphate-in medium and long-chain triglyceride, respectively, and 25.3% and 80.8% in modified and un-modified kaolinite nanoparticle-stabilized Pickering emulsions (Shah et al., 2016b, Tang et al., 2019, Xiao et al., 2015). This confirms an improved effect of the Pickering emulsion systems designed in this study on the bioaccessibility of CUR over previous studies and thus supports the hypothesis that effective particle design and Pickering emulsion formation can enhance the total amount of CUR that can be made available into the micellar phase after digestion. Interestingly, there were no clear relationship between improved gastric stability of the emulsions and bioaccessibility of CUR. In other words, gastric-stable emulsions with complex interfaces (CUR-DxS+E_WPN_, CUR-E_WPDxM_) were not advantageous over the simple nanogel-particles (CUR-E_WPN_) in terms of bioaccessibility.

### 3.4 Cell viability and uptake in the presence of CUR

**Fig. 6:**
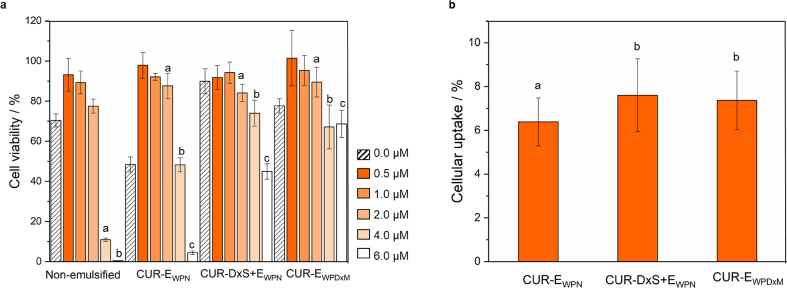
Cell viability (a) of MCT-dissolved CUR (non-emulsified) systems and the three Pickering emulsion-based delivery vehicles (CUR-E_WPN_, CUR-DxS+E_WPN_ and CUR-E_WPDxM_) at different concentrations of CUR against Caco-2 cells incubated for 2 h along with the digested blank emulsions without any curcumin (blank bars with diagonal lines), and cellular uptake (b) of CUR by the Caco-2 cells in the three Pickering emulsion-based delivery vehicles. Data presented are mean with standard deviation of three independent experiments. Different letters indicate mean significant differences between CUR concentrations (0.5 μM - μM) for cell viability, and significant diferences between emulsion types (CUR-E_WPN_, CUR-DxS-E_WPN_ and CUR-E_WPDxM_) for cellular uptake.

Next, we aimed to understand whether the increased gastric stability of the emulsions designed with complex interfaces had any impact on the viability of Caco-2 cells and benefit in terms of cellular CUR uptake. From Fig. 6a, it can be observed that CUR-encapsulated Pickering emulsion formulations, as well as CUR concentration, had a significant effect on cell viability of Caco-2 cells. It has been previously reported that incubation of bile acids with Caco-2 cells reversibly decreases the molecular diffusion across the intestinal epithelium (Münch et al., 2007, Raimondi et al., 2008). Hence, a control experiment was conducted to evaluate the toxicity of bile salts in the *in vitro* digestion medium (*i.e.* SGF + SIF) (Supplementary Fig. S1). In addition, CUR in non-emulsified MCT-oil (Fig. 6a) and digested Pickering emulsions without added CUR (blank) were also investigated as controls (Fig. 6a). Digested blank-Pickering emulsions without CUR exhibited some cytotoxicity to the cells in E_WPN_ and E_WPDxM_ systems with cell viability below 80% at an equivalent CUR concentration of 0.50 μM (Fig. 6a). This might be attributed to the gradual decrease in cell viability with increasing bile salt concentration (Supplementary Fig. S1) which suggests that possibly the digestion medium (SGF and SIF) resulted in some degree of cytotoxicity, which might be attributed to bile salt-mediated disruption of lipidic cell membranes via its surfactant-like activity and consequently necrosis and cellular injury (Perez and Briz, 2009).

In Fig. 6a, it is shown that more than 80% cell viability was retained for all Pickering emulsion systems from 0.5 to 2 μM CUR concentration after 2 h of incubation but was below 80% for the non-emulsified sample at 2 μM. With increasing CUR concentration to 4 and 6 μM, the viability of Caco-2 cells decreased to below 80%, especially for the non-emulsified and CUR-E_WPN_, which clearly suggests that CUR-DxS+E_WPN_ and CUR-E_WPDxM_ were significantly less toxic at higher CUR concentrations (4 and 6 μM) as compared to non-emulsified CUR dissolved in MCT oil. In other words, Pickering emulsion systems with complex interfaces were more effective in reducing digestion-medium associated alteration of the encapsulated CUR and consequently toxicity to the Caco-2 cells. Given the effects of CUR on cell viability, a concentration of 1 μM was used for the cellular uptake study.

As shown in Fig. 6b, the use of a biopolymer by complexation significantly increased the cellular uptake from 6.3 ± 1.09% for CUR-E_WPN_ to 7.6 ± 1.66% for CUR-DxS+E_WPN_, whereas conjugation did not increase the cellular uptake (7.36 ± 1.34% for CUR-E_WPDxM_) significantly. Previous literature that has assessed the cellular uptake of CUR in Caco-2 cells after *in vitro* digestion have reported an increased cellular uptake from 4.44 ± 0.11 to 10.5 ± 0.15% when using whey protein isolate and whey protein isolate-coated with chitosan nanoemulsions, respectively (Silva et al., 2019). Other studies have recently demonstrated intracellular uptake in Caco-2 cells of CUR encapsulated in different nanocarriers such as polymer micelles, nanoemulsion and liposome (Yan et al., 2019). The maximum cellular uptake reported for these systems at a CUR concentration of 100 μM was 3.67 ± 0.10, 5.55 ± 0.13, 3.36 ± 0.51 and 6.46 ± 0.18% for free CUR, polymer micelles, liposomes, and nanoemulsions, respectively. In this study by Yan et al. (2019), the increased uptake was attributed to the positively-charged multilayer nanoemulsion, which was hypothesized to be more effectively internalized into the Caco-2 cells.

Our results suggest that the cellular uptake for CUR encapsulated in Pickering emulsions was significantly increased as compared to free CUR, polymer micelles, liposomes and nanoemulsions reported in previous studies and that DxS-coated nanogel-stabilized or Dx-conjugated microgel-stabilized Pickering emulsions (7.4–7.6% uptake) were more effective vehicles to deliver CUR to Caco-2 cells. This might be explained by an increase in colonic mucosal permeability caused by dextran (Kitajima et al., 1999). In addition, it is worth reminding that both these emulsions with complex interfaces were gastric stable and offered increased cell viability. Therefore, at this stage, we hypothesize that reduced physiological degradation of CUR in the gastric phase (Kharat et al., 2017, Tønnesen and Karlsen, 1985, Wang et al., 1997) obtained by gastric-stable emulsions using complex particle-biopolymer interfaces, can be a potential mechanism contributing to the reduced cellular toxicity at an increased concentration of CUR and enhanced cellular internalization of CUR, however, the exact mechanism needs further investigation in the future.

## 4 Conclusions

The purpose of this study was to evaluate Pickering emulsion systems with complex interfaces for delivery, bioaccessibility and cellular uptake of curcumin after *in vitro* simulated gastric and small intestinal digestion phase. Results show that all curcumin-loaded Pickering emulsions systems can increase the bioaccessibility of curcumin as compared to non-emulsified curcumin dissolved in bulk oil. Also, curcumin-loaded Pickering emulsions were significantly less toxic at higher curcumin concentrations as compared to non-emulsified curcumin dissolved in bulk oil, indicating the importance of delivering curcumin using a delivery vehicle. Cellular uptake results showed that Pickering emulsions with complex interfaces that provided kinetic stability to coalescence in the gastric conditions can enhance the cellular uptake of curcumin in Caco-2 cells. This study suggests that the development of Pickering emulsions with suitable interfacial engineering can be used as effective templates to increase bioaccessibility and cellular uptake of curcumin. Further studies are needed to clearly understand the mechanism behind better cellular internalization of curcumin in the Pickering emulsions designed with complex interfaces and whether or not gastric stability has a direct correlation with cell viability.

## CRediT authorship contribution statement

**Andrea Araiza-Calahorra:** Writing - original draft, Methodology, Validation, Formal analysis, Investigation, Data curation, Writing - review & editing, Visualization, Project administration, Funding acquisition. **Yunqing Wang:** Formal analysis, Investigation. **Christine Boesch:** Methodology, Formal analysis, Writing - review & editing, Validation. **Yansheng Zhao:** Methodology, Formal analysis, Data curation, Writing - review & editing. **Anwesha Sarkar:** Methodology, Validation, Conceptualization, Data curation, Writing - review & editing, Visualization, Supervision.

## Declaration of Competing Interest

The authors declare no conflicts of interests.

## References

[bib1] Amani S., Mohamadnia Z., Mahdavi A. (2019). pH-responsive hybrid magnetic polyelectrolyte complex based on alginate/BSA as efficient nanocarrier for curcumin encapsulation and delivery. Int. J. Biol. Macromol..

[bib2] Anand P., Kunnumakkara A.B., Newman R.A., Aggarwal B.B. (2007). Bioavailability of curcumin: problems and promises. Mol. Pharm..

[bib4] Araiza-Calahorra A., Sarkar A. (2019). Pickering emulsion stabilized by protein nanogel particles for delivery of curcumin: effects of pH and ionic strength on curcumin retention. Food Struct..

[bib3] Araiza-Calahorra A., Sarkar A. (2019). Designing biopolymer-coated Pickering emulsions to modulate in vitro gastric digestion: a static model study. Food Funct..

[bib5] Araiza-Calahorra A., Akhtar M., Sarkar A. (2018). Recent advances in emulsion-based delivery approaches for curcumin: from encapsulation to bioaccessibility. Trends Food Sci. Technol..

[bib6] Araiza-Calahorra A., Glover Z.J., Akhtar M., Sarkar A. (2020). Conjugate microgel-stabilized Pickering emulsions: role in delaying gastric digestion. Food Hydrocolloids.

[bib7] Asabuwa Ngwabebhoh F., Ilkar Erdagi S., Yildiz U. (2018). Pickering emulsions stabilized nanocellulosic-based nanoparticles for coumarin and curcumin nanoencapsulations: in vitro release, anticancer and antimicrobial activities. Carbohydr. Polym..

[bib8] Destribats M., Rouvet M., Gehin-Delval C., Schmitt C., Binks B.P. (2014). Emulsions stabilised by whey protein microgel particles: towards food-grade Pickering emulsions. Soft Matter.

[bib9] Dickinson E. (2012). Use of nanoparticles and microparticles in the formation and stabilization of food emulsions. Trends Food Sci. Technol..

[bib10] Espinal-Ruiz M., Parada-Alfonso F., Restrepo-Sánchez L.P., Narváez-Cuenca C.E., McClements D.J. (2014). Impact of dietary fibers [methyl cellulose, chitosan, and pectin] on digestion of lipids under simulated gastrointestinal conditions. Food Funct..

[bib11] Goel A., Kunnumakkara A.B., Aggarwal B.B. (2008). Curcumin as “Curecumin”: from kitchen to clinic. Biochem. Pharmacol..

[bib12] Kharat M., McClements D.J. (2019). Recent advances in colloidal delivery systems for nutraceuticals: a case study – delivery by design of curcumin. J. Colloid Interface Sci..

[bib13] Kharat M., Du Z., Zhang G., McClements D.J. (2017). Physical and chemical stability of curcumin in aqueous solutions and emulsions: impact of pH, temperature, and molecular environment. J. Agric. Food Chem..

[bib14] Kitajima S., Takuma S., Morimoto M. (1999). Changes in colonic mucosal permeability in mouse colitis induced with dextran sulfate sodium. Exp. Anim..

[bib15] Kolter M., Wittmann M., Köll-Weber M., Süss R. (2019). The suitability of liposomes for the delivery of hydrophobic drugs – a case study with curcumin. Eur. J. Pharm. Biopharm..

[bib16] Lu X., Li C., Huang Q. (2019). Combining in vitro digestion model with cell culture model: assessment of encapsulation and delivery of curcumin in milled starch particle stabilized Pickering emulsions. Int. J. Biol. Macromol..

[bib17] Marefati A., Bertrand M., Sjöö M., Dejmek P., Rayner M. (2017). Storage and digestion stability of encapsulated curcumin in emulsions based on starch granule Pickering stabilization. Food Hydrocolloids.

[bib18] Meshulam D., Lesmes U. (2014). Responsiveness of emulsions stabilized by lactoferrin nano-particles to simulated intestinal conditions. Food Funct..

[bib19] Minekus M., Alminger M., Alvito P., Ballance S., Bohn T., Bourlieu C., Carrière F., Boutrou R., Corredig M., Dupont D., Dufour C., Egger L., Golding M., Karakaya S., Kirkhus B., Le Feunteun S., Lesmes U., Macierzanka A., Mackie A., Marze S., McClements D.J., Ménard O., Recio I., Santos C.N., Singh R.P., Vegarud G.E., Wickham M.S.J., Weitschies W., Brodkorb A. (2014). A standardised static in vitro digestion method suitable for food – an international consensus. Food Funct..

[bib20] Münch A., Ström M., Söderholm J.D. (2007). Dihydroxy bile acids increase mucosal permeability and bacterial uptake in human colon biopsies. Scand. J. Gastroenterol..

[bib21] Perez M.J., Briz O. (2009). Bile-acid-induced cell injury and protection. World J. Gastroenterol..

[bib22] Perez-Hernandez L.M., Nugraheni K., Benohoud M., Sun W., Hernández-Álvarez A.J., Morgan M.R.A., Boesch C., Orfila C. (2020). Starch digestion enhances bioaccessibility of anti-inflammatory polyphenols from Borlotti Beans (Phaseolus vulgaris). Nutrients.

[bib23] Raimondi F., Santoro P., Barone M.V., Pappacoda S., Barretta M.L., Nanayakkara M., Apicella C., Capasso L., Paludetto R. (2008). Bile acids modulate tight junction structure and barrier function of Caco-2 monolayers via EGFR activation.

[bib24] Rayner M., Sjöö M., Timgren A., Dejmek P. (2012). Quinoa starch granules as stabilizing particles for production of Pickering emulsions. Faraday Discuss.

[bib25] Ruiz-Rodriguez P.E., Meshulam D., Lesmes U. (2014). Characterization of pickering O/W emulsions stabilized by silica nanoparticles and their responsiveness to in vitro digestion conditions. Food Biophys..

[bib26] Salvia-Trujillo L., Qian C., Martín-Belloso O., McClements D.J. (2013). Influence of particle size on lipid digestion and β-carotene bioaccessibility in emulsions and nanoemulsions. Food Chem..

[bib27] Salvia-Trujillo L., Qian C., Martín-Belloso O., McClements D.J. (2013). Modulating β-carotene bioaccessibility by controlling oil composition and concentration in edible nanoemulsions. Food Chem..

[bib28] Sarkar A., Mackie A.R. (2020). Engineering oral delivery of hydrophobic bioactives in real-world scenarios. Curr. Opin. Colloid Interface Sci..

[bib29] Sarkar A., Murray B., Holmes M., Ettelaie R., Abdalla A., Yang X. (2016). In vitro digestion of Pickering emulsions stabilized by soft whey protein microgel particles: influence of thermal treatment. Soft Matter.

[bib30] Sarkar A., Zhang S., Murray B., Russell J.A., Boxal S. (2017). Modulating in vitro gastric digestion of emulsions using composite whey protein-cellulose nanocrystal interfaces. Colloids Surf. B Biointerfaces.

[bib32] Sarkar A., Zhang S., Holmes M., Ettelaie R. (2019). Colloidal aspects of digestion of Pickering emulsions: experiments and theoretical models of lipid digestion kinetics. Adv. Colloid Interface Sci..

[bib33] Shah B.R., Li Y., Jin W., An Y., He L., Li Z., Xu W., Li B. (2016). Preparation and optimization of Pickering emulsion stabilized by chitosan-tripolyphosphate nanoparticles for curcumin encapsulation. Food Hydrocolloids.

[bib34] Shah B.R., Zhang C., Li Y., Li B. (2016). Bioaccessibility and antioxidant activity of curcumin after encapsulated by nano and Pickering emulsion based on chitosan-tripolyphosphate nanoparticles. Food Res. Int..

[bib35] Shimoni G., Shani Levi C., Levi Tal S., Lesmes U. (2013). Emulsions stabilization by lactoferrin nano-particles under in vitro digestion conditions. Food Hydrocolloids.

[bib36] Silva H.D., Beldíková E., Poejo J., Abrunhosa L., Serra A.T., Duarte C.M.M., Brányik T., Cerqueira M.A., Pinheiro A.C., Vicente A.A. (2019). Evaluating the effect of chitosan layer on bioaccessibility and cellular uptake of curcumin nanoemulsions. J. Food Eng..

[bib37] Singh H., Sarkar A. (2011). Behaviour of protein-stabilised emulsions under various physiological conditions. Adv. Colloid Interface Sci..

[bib38] Tang Q., Xie X., Li C., Zhen B., Cai X., Zhang G., Zhou C., Wang L. (2019). Medium-chain triglyceride/water Pickering emulsion stabilized by phosphatidylcholine-kaolinite for encapsulation and controlled release of curcumin. Colloids Surf. B Biointerfaces.

[bib39] Tikekar R.V., Pan Y., Nitin N. (2013). Fate of curcumin encapsulated in silica nanoparticle stabilized Pickering emulsion during storage and simulated digestion. Food Res. Int..

[bib41] Tønnesen H.H., Karlsen J. (1985). Studies on curcumin and curcuminoids. VI. Kinetics of curcumin degradation in aqueous solution. Z. Lebensm. Unters. Forsch..

[bib42] Tønnesen H.H., Másson M., Loftsson T. (2002). Studies of curcumin and curcuminoids. XXVII. Cyclodextrin complexation: solubility, chemical and photochemical stability. Int. J. Pharm..

[bib43] Torres O., Murray B.S., Sarkar A. (2019). Overcoming in vitro gastric destabilisation of emulsion droplets using emulsion microgel particles for targeted intestinal release of fatty acids. Food Hydrocolloids.

[bib44] Tzoumaki M.V., Moschakis T., Kiosseoglou V., Biliaderis C.G. (2011). Oil-in-water emulsions stabilized by chitin nanocrystal particles. Food Hydrocolloids.

[bib45] Wang Y.-J., Pan M.-H., Cheng A.-L., Lin L.-I., Ho Y.-S., Hsieh C.-Y., Lin J.-K. (1997). Stability of curcumin in buffer solutions and characterization of its degradation products. J. Pharmaceut. Biomed. Anal..

[bib46] Wei Z., Zhu J., Cheng Y., Huang Q. (2019). Ovotransferrin fibril–stabilized Pickering emulsions improve protection and bioaccessibility of curcumin. Food Res. Int..

[bib47] Xiao J., Li C., Huang Q. (2015). Kafirin nanoparticle-stabilized pickering emulsions as oral delivery vehicles: physicochemical stability and in vitro digestion profile. J. Agric. Food Chem..

[bib48] Yan X., Cao S., Li Y., Xiao P., Huang Z., Li H., Ma Y. (2019). Internalization and subcellular transport mechanisms of different curcumin loaded nanocarriers across Caco-2 cell model. J. Drug Deliv. Sci. Technol..

[bib49] Zou L., Zheng B., Liu W., Liu C., Xiao H., McClements D.J. (2015). Enhancing nutraceutical bioavailability using excipient emulsions: influence of lipid droplet size on solubility and bioaccessibility of powdered curcumin. J. Funct. Foods.

