# Supplementary material for: Pickering emulsions stabilized by colloidal gel particles complexed or conjugated with biopolymers to enhance bioaccessibility and cellular uptake of curcumin
Source: Curr Res Food Sci. 2020 May 13;3:178–88. doi: 10.1016/j.crfs.2020.05.001 (PMC7473359; doi:10.1016/j.crfs.2020.05.001)
Supplement: Supplementary file 1 — Multimedia component 1 [file mmc1.docx]

**Supplementary Information**

**Pickering emulsions stabilized by colloidal gel particles complexed or conjugated with biopolymers to enhance bioaccessibility and cellular uptake of curcumin**

**Andrea Araiza-Calahorra^a^, Yunqing Wang^b^, Christine Boesch^b^, Yansheng Zhao^c^** and **Anwesha Sarkar^a*^**

^a^ *Food Colloids and Bioprocessing Group, School of Food Science and Nutrition, University of Leeds, Leeds, LS2 9JT, UK*

^b^ *Nutritional Sciences and Epidemiolgy Group, School of Food Science and Nutrition, University of Leeds, Leeds, LS2 9JT, UK*

^c^ *School of Food and Biological Engineering, Jiangsu University, Zhenjiang, 212013, China*

Corresponding author email*: [A.Sarkar@leeds.ac.uk](mailto:A.Sarkar@leeds.ac.uk) (A. Sarkar).


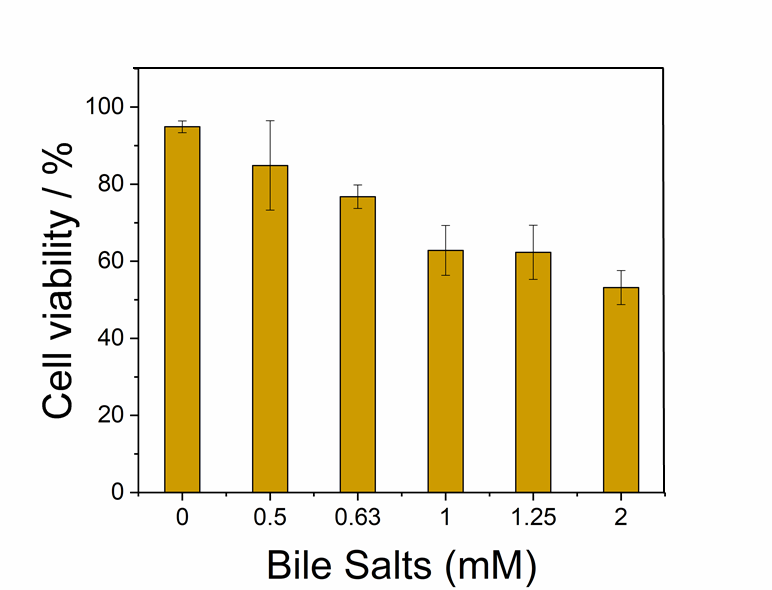


**Figure S1.** Cytotoxicity of bile salts towards Caco-2 cells following a 2 h incubation in the simulated digestion medium (i.e. simulated gastric and intestinal fluids without emulsion and CUR). Data are mean with SD from three independent experiments performed in duplicate.
